# The Role of Iron in Prion Disease and Other Neurodegenerative Diseases

**DOI:** 10.1371/journal.ppat.1004335

**Published:** 2014-09-18

**Authors:** Neena Singh

**Affiliations:** Department of Pathology, Case Western Reserve University, Cleveland, Ohio, United States of America; Washington University School of Medicine, United States of America

A growing number of neurodegenerative conditions, such as sporadic Creutzfeldt-Jakob disease (sCJD), Alzheimer's disease (AD), and Parkinson's disease (PD), are associated with accumulation of iron in the brain. The underlying cause is complex and only partially known. A better understanding of whether iron plays a primary or secondary role in disease pathogenesis is desirable and is likely to help in the development of disease-specific therapeutic strategies.

## Is Brain Iron Dyshomeostasis Primary or Secondary to Neurodegeneration?

Iron is essential for vital metabolic processes, but unshielded iron is toxic via Fenton chemistry. Iron metabolism is, therefore, tightly regulated to maintain homeostasis at the cellular, organelle, and systemic levels. Nonetheless, iron accumulates in several brain disorders. The mechanism underlying iron-induced neurotoxicity is evident in disorders where the function of the pathogenic protein in cellular iron metabolism is known, and consequences of dysfunction or absence are predictable, as in hereditary ferritinopathy and aceruloplasminemia. Ambiguity arises for neurodegenerative conditions, such as sCJD, AD, and PD, where brain iron dyshomeostasis is associated with aggregation of proteins with poorly defined function [1]. The diverse etiology and pathophysiology of these disorders suggests that iron accumulation follows neuronal death and is therefore of little therapeutic significance. Emerging data, however, requires a reconsideration of this concept. First, principal proteins implicated in the pathogenesis of sCJD, AD, and PD participate in cellular iron metabolism, and their aggregation induces specific changes in the expression of iron modulating proteins in the brain and cerebrospinal fluid (CSF), some reaching diagnostic significance [2], [3]. Second, sequestration of iron in aggregated protein complexes induces functional iron deficiency despite increased redox-active iron, escalating the neurotoxicity [4], [5]. Lastly, a coping response from surviving neurons worsens the iron dyshomeostasis and associated neurotoxicity [4].

An additional layer of complexity is introduced by the secondary effects of inflammation, microgliosis, and astrocytosis that follow neuronal insult, each causing distinct changes in brain iron metabolism through specific pathways. The distinction between primary and secondary events blurs with disease progression, fueling the controversy on whether brain iron dyshomeostasis is primary or secondary to disease pathogenesis [6].

## How Does the Brain Maintain Iron Homeostasis?

**Figure 1:**
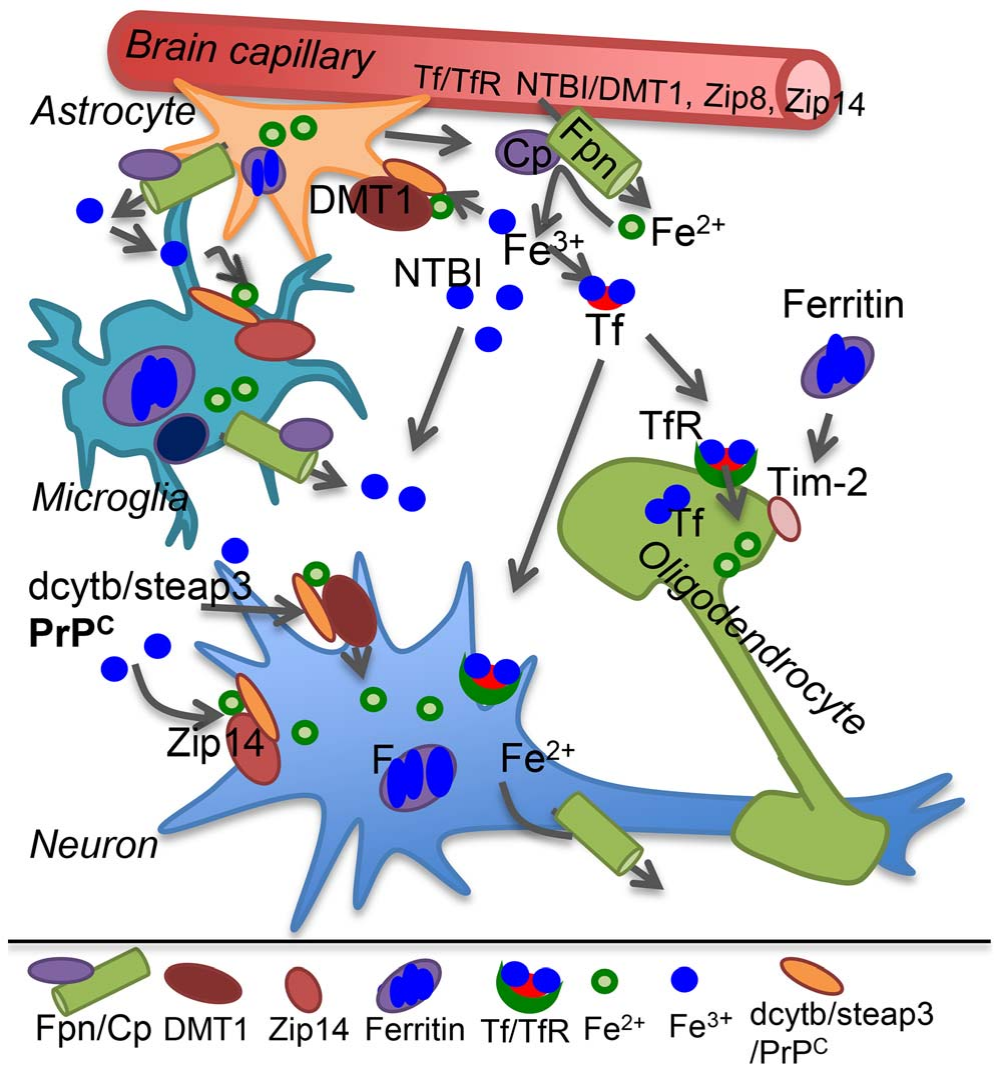
Iron transport in the brain. Plasma Tf-iron is taken up by the Tf/TfR pathway, and NTBI by the metal transporters DMT1, Zip8, or Zip14 on the apical plasma membrane of capillary endothelial cells and transported across the basolateral membrane to brain interstitial fluid and CSF by the coupled action of Fpn and the ferroxidase ceruloplasmin (Cp) secreted by astrocytes. Cp oxidizes Fe^2+^ to Fe^3+^ that binds CSF Tf or circulates as NTBI. Astrocytes lack TfR and utilize NTBI that requires reduction to Fe^2+^ by FR proteins dcytb, steap3, or PrP^C^ before uptake by metal transporters. Microglia store iron released from phagocytosed cells and excess NTBI in the extracellular milieu in ferritin. Neurons take up Tf-iron by the Tf/TfR pathway, and NTBI through metal transporters as in astrocytes. Oligodendrocytes synthesize and utilize Tf-iron and also take up ferritin iron by the Tim-2 receptor. Internalized iron contributes to the cytosolic labile iron pool for metabolic purposes and excess is oxidized and stored in ferritin. Most cells express Fpn, and export excess Fe^2+^ through the coupled action of Fpn and Cp. Cp: ceruloplasmin; DMT1: divalent metal transporter 1; F: ferritin; Fpn: ferroportin; FR: ferrireductase; NTBI: non-transferrin bound iron; Tf: transferrin; TfR: transferrin receptor.

All brain cells are bathed in CSF that provides iron and other nutrients necessary for survival. Transport of transferrin (Tf)-iron and non-Tf-bound iron (NTBI) from the systemic circulation to the CSF across the blood brain barrier (BBB) is mediated by the transferrin receptor (TfR) and metal transporters respectively. Both Tf-iron and NTBI are in the relatively nonreactive oxidized (Fe^3+^) form, requiring reduction to Fe^2+^ by ferrireductase (FR) proteins for transport across lipid bilayers. Known FR proteins include duodenal cytochrome b (dcytb), steap 2 and 3, and the prion protein (PrP^C^) [1], [7], [8]. Known metal transporters include divalent metal transporter-1 (DMT1), Zip8, and Zip14 [1]. Transported Fe^2+^ joins the labile iron pool (LIP) of endothelial cells, is oxidized and stored by cellular ferritin, or is exported to the CSF through the coupled action of ferroportin (Fpn) and ceruloplasmin that oxidizes released Fe^2+^ to Fe^3+^ that binds CSF Tf or circulates as NTBI (Figure 1) [1].

Neurons take up both Tf-iron and NTBI, astrocytes lack TfR and utilize only NTBI, and oligodendrocytes acquire iron from Tf and ferritin. Microglia recycle iron from phagocytosed cells and internalize excess NTBI for storage (Figure 1). Individual cells maintain iron homeostasis by the coordinated effort of iron regulatory proteins (IRPs) that bind to iron responsive elements (IREs) on the transcripts of iron uptake and storage proteins, modulating their stability and translation based on cellular iron needs. Iron deficiency causes up-regulation of Tf, TfR, and DMT1, and down-regulation of ferritin. The converse occurs in iron overload [1]. This mechanism ensures iron availability without any unshielded Fe^2+^ and is especially important in the brain since iron saturation of CSF Tf is 100% as opposed to ∼30% for serum Tf, leaving little buffering capacity against excess iron.

## Are Prion Disorders Associated with Brain Iron Dyshomeostasis?

Prion disorders result from the change in conformation of prion protein (PrP^C^) to a misfolded PrP-scrapie (PrP^Sc^) form that accumulates in the brain parenchyma. Brain iron dyshomeostasis is a prominent feature of human and animal prion disorders and has been reported by several laboratories using unrelated techniques and experimental models. Biochemical analysis of brain tissue from sCJD and scrapie-infected mouse and hamster models shows increased reactivity for redox-active iron and, paradoxically, a phenotype of neuronal iron deficiency [4], [9]. Pre-mortem CSF from sCJD cases reflects these changes, allowing discrimination from other dementias with a high degree of sensitivity and specificity [2]. A systems biology approach reveals differential expression of genes involved in iron homeostasis and heme metabolism in scrapie-infected mouse brains [10], and a molecular biology approach indicates altered expression of iron regulatory and storage proteins in distinct brain regions of scrapie-infected mice [11]. Alteration of iron metabolism is also noted in the spleen of scrapie-infected mice, the main peripheral organ that replicates and accumulates peripherally introduced PrP^Sc^ [12]. Likewise, cell lines replicating PrP^Sc^ in culture show altered expression of iron regulatory and storage proteins, supporting the in vivo observations [13]. Moreover, exposure to redox-active iron, hemin, or copper causes aggregation of cell-associated and recombinant PrP^C^ to a detergent-insoluble form that, in some cases, induces aggregation of additional PrP^C^ [14], suggesting that, once initiated, brain iron dyshomeostasis is likely to escalate prion disease pathogenesis by more than one pathway.

## What Is the Cause of Brain Iron Dyshomeostasis in Prion Disorders?

Cumulative evidence suggests that accumulation of iron in PrP^Sc^ aggregates combined with loss of function of PrP^C^ in iron uptake contributes to the iron dyshomeostasis in prion disease affected brains. Thus, scrapie-infected mouse and hamster brain and spinal cord tissue show a phenotype of iron deficiency that increases with disease progression and correlates with PrP^Sc^, indicating a causal relationship [4], [9]. The likely cause is sequestration of iron in PrP^Sc^–ferritin aggregates in a biologically unavailable form in the lysosomes of scrapie-infected cells [4], [14]. These aggregates are relatively stable and co-purify from infected brain homogenates despite harsh purification conditions [4], [15]. Like PrP^Sc^, ferritin from diseased brains resists protease digestion, is insoluble in detergents, and retains associated iron despite denaturation by heat and detergents [9]. Chelation of iron increases the sensitivity of PrP^Sc^ to protease digestion, indicating that iron stabilizes the structure of PrP^Sc^ [14]. Notably, PrP^Sc^ and ferritin are co-transported across intestinal epithelial cells in vitro and in vivo, suggesting clinical implications of PrP^Sc^–ferritin aggregates beyond brain iron dyshomeostasis [15].

Additionally, conversion of PrP^C^ to PrP^Sc^ is likely to compromise its functional activity in cellular iron uptake, worsening the iron deficiency in diseased brains. PrP^C^ promotes iron uptake by providing the necessary FR activity [7], and its absence in PrP^−/−^ mice reduces the iron content of major systemic organs, hematopoietic cells, and the brain [16]. Uptake of NTBI is affected more than Tf-iron as indicated by decreased iron content of liver and bone marrow macrophages of PrP^−/−^ mice despite experimentally-induced systemic iron overload [7]. Reabsorption of NTBI from the glomerular filtrate is also reduced in PrP^−/−^ mice, suggesting a prominent role of PrP^C^ in kidney iron metabolism (unpublished observations). Introduction of PrP^C^ in the PrP^−/−^ background restores iron metabolism to wild-type levels, underscoring the functional role of PrP^C^ in iron uptake [16]. Altered levels of iron in the brains of PrP^−/−^, wild type, and PrP-overexpressing mice as a function of PrP^C^ expression have also been reported using X-ray fluorescence imaging, supporting these observations [17].

## What Is the Cause of Brain Iron Dyshomeostasis in AD and PD?

Regional accumulation of iron has been reported in AD and PD brains and results from distinct pathogenic processes [1]. In AD, iron accumulates in amyloid plaques in association with amyloid beta (Aβ), a proteolytic product of the amyloid precursor protein (APP). Aβ binds iron with high affinity, rendering it redox-active. This causes localized accumulation of iron and aggregation of additional Aβ, amplifying the process [5]. Moreover, APP mediates the export of excess neuronal iron by stabilizing Fpn and providing the necessary ferroxidase activity and is itself regulated by cellular iron through IRE sequences in its transcript [18], [19]. The development of brain iron dyshomeostasis in AD is therefore complex and involves altered functional activity of APP, sequestration of iron in Aβ aggregates, and additional processes discussed in a recent review [19]. A full understanding of the underlying mechanism, however, is lacking.

PD has a multifactorial etiology and is associated with loss of dopamine (DA)-producing neurons in the substantia nigra (SN). Accumulation of iron in PD brains has been attributed to secondary causes such as mitochondrial dysfunction and release from iron-rich DA neurons. However, up-regulation of DMT1 and down-regulation of Fpn in the SN of human cases and experimental models of PD suggests that accumulation of iron in PD is an active process, not a mere outcome of disease pathogenesis, and requires further exploration [20].

**Figure 2:**
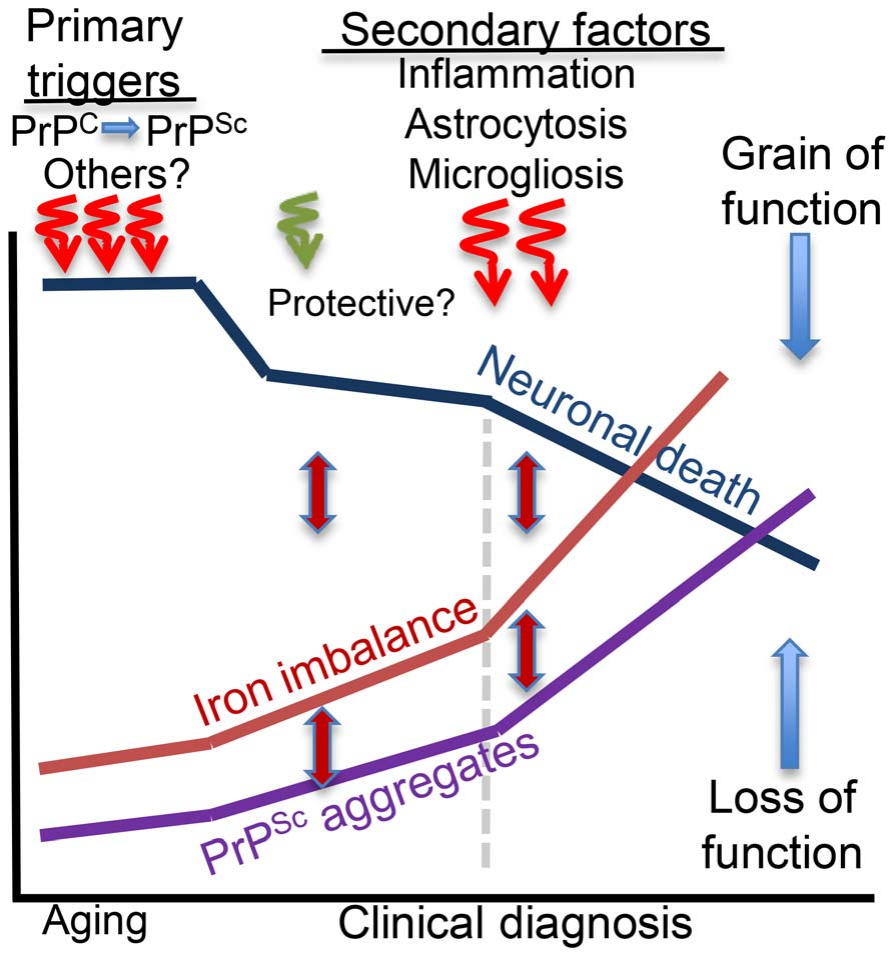
Hypothetical model of brain iron dyshomeostasis in neurodegenerative disorders. Primary triggers of neurotoxicity include dysfunction or aggregation of the protein implicated in the pathogenesis of a specific neurodegenerative condition and other less defined factors including aging. Microglia and astrocytes respond to neuronal death and mount a protective response, which is soon overwhelmed by the accumulation of redox-active protein aggregates that induce neurotoxicity and accumulation of additional protein aggregates, increase in brain iron dyshomeostasis, and oxidative stress. These responses are inter-linked, and form a vicious cycle. Inflammatory response to neuronal death increases cytokine release that activates astrocytes and increases microglial activity and death with the release of intracellular iron. These events increase iron imbalance, redox-iron mediated protein aggregation, and significantly increase neurotoxicity. Brain iron dyshomeostasis occurs early and is one of the primary triggers of neurotoxicity if the pathogenic protein is involved in cellular iron metabolism. In other cases, iron dyshomeostasis is initiated by neuronal death, is fueled by microgliosis, activated astrocytes, and redox-active protein aggregates, and spirals when inflammation sets in, amplifying the neurotoxicity.

In conclusion, primary triggers that induce brain iron dyshomeostasis in sCJD, AD, and PD are specific to each disorder. Secondary events that follow neuronal injury are shared, each contributing to iron dyshomeostasis through distinct pathways (Figure 2). Ideally, therapeutic strategies should inhibit or block both processes and reduce iron-induced neurotoxicity by restoring brain iron homeostasis. This is a challenging task because most neurodegenerative conditions are initiated by multiple events, including aging, and are fuelled by several pathways that converge to neuronal demise. Iron dyshomeostasis is one pathway in this continuum of events that holds therapeutic promise and requires focused investigation.

## References

[1] Singh N, Haldar S, Tripathi AK, Horback K, Wong J, et al (2014) Brain iron homeostasis: from molecular mechanisms to clinical significance and therapeutic opportunities. Antioxid Redox Signal 20: 1324–1363. 23815406 10.1089/ars.2012.4931 PMC3935772

[2] Haldar S, Beveridge J, Wong J, Singh A, Galimberti D, et al (2013) A low-molecular-weight ferroxidase is increased in the CSF of sCJD cases: CSF ferroxidase and transferrin as diagnostic biomarkers for sCJD. Antioxid Redox Signal 19: 1662–1675. 23379482 10.1089/ars.2012.5032 PMC3809602

[3] Olivieri S, Conti A, Iannaccone S, Cannistraci CV, Campanella A, et al (2011) Ceruloplasmin oxidation, a feature of Parkinson's disease CSF, inhibits ferroxidase activity and promotes cellular iron retention. J Neurosci 31: 18568–18577. 22171055 10.1523/JNEUROSCI.3768-11.2011 PMC6623910

[4] Singh A, Isaac AO, Luo X, Mohan ML, Cohen ML, et al (2009) Abnormal brain iron homeostasis in human and animal prion disorders. PLoS Pathog 5: e1000336. 19283067 10.1371/journal.ppat.1000336 PMC2652663

[5] Greenough MA, Camakaris J, Bush AI (2013) Metal dyshomeostasis and oxidative stress in Alzheimer's disease. Neurochem Int 62: 540–555. 22982299 10.1016/j.neuint.2012.08.014

[6] Andersen HH, Johnsen KB, Moos T (2014) Iron deposits in the chronically inflamed central nervous system and contributes to neurodegeneration. Cell Mol Life Sci 71: 1607–1622. 24218010 10.1007/s00018-013-1509-8 PMC3983878

[7] Singh A, Haldar S, Horback K, Tom C, Zhou L, et al (2013) Prion protein regulates iron transport by functioning as a ferrireductase. J Alzheimers Dis 35: 541–552. 23478311 10.3233/JAD-130218 PMC5450724

[8] Ohgami RS, Campagna DR, McDonald A, Fleming MD (2006) The Steap proteins are metalloreductases. Blood 108: 1388–1394. 16609065 10.1182/blood-2006-02-003681 PMC1785011

[9] Singh A, Qing L, Kong Q, Singh N (2012) Change in the characteristics of ferritin induces iron imbalance in prion disease affected brains. Neurobiol Dis 45: 930–938. 22182691 10.1016/j.nbd.2011.12.012 PMC3286598

[10] Hwang D, Lee IY, Yoo H, Gehlenborg N, Cho JH, et al (2009) A systems approach to prion disease. Mol Syst Biol 5: 252. 19308092 10.1038/msb.2009.10 PMC2671916

[11] Kim BH, Jun YC, Jin JK, Kim JI, Kim NH, et al (2007) Alteration of iron regulatory proteins (IRP1 and IRP2) and ferritin in the brains of scrapie-infected mice. Neurosci Lett 422: 158–163. 17614197 10.1016/j.neulet.2007.05.061 PMC2365884

[12] Huzarewich RL, Medina S, Robertson C, Parchaliuk D, Booth SA (2011) Transcriptional modulation in a leukocyte-depleted splenic cell population during prion disease. J Toxicol Environ Health A 74: 1504–1520. 22043911 10.1080/15287394.2011.618979

[13] Fernaeus S, Halldin J, Bedecs K, Land T (2005) Changed iron regulation in scrapie-infected neuroblastoma cells. Brain Res Mol Brain Res 133: 266–273. 15710243 10.1016/j.molbrainres.2004.10.018

[14] Basu S, Mohan ML, Luo X, Kundu B, Kong Q, et al (2007) Modulation of proteinase K-resistant prion protein in cells and infectious brain homogenate by redox iron: implications for prion replication and disease pathogenesis. Mol Biol Cell 18: 3302–3312. 17567949 10.1091/mbc.E07-04-0317 PMC1951779

[15] Mishra RS, Basu S, Gu Y, Luo X, Zou WQ, et al (2004) Protease-resistant human prion protein and ferritin are cotransported across Caco-2 epithelial cells: implications for species barrier in prion uptake from the intestine. J Neurosci 24: 11280–11290. 15601934 10.1523/JNEUROSCI.2864-04.2004 PMC6730364

[16] Singh A, Kong Q, Luo X, Petersen RB, Meyerson H, et al (2009) Prion protein (PrP) knock-out mice show altered iron metabolism: a functional role for PrP in iron uptake and transport. PLoS ONE 4: e6115. 19568430 10.1371/journal.pone.0006115 PMC2699477

[17] Pushie MJ, Pickering IJ, Martin GR, Tsutsui S, Jirik FR, et al (2011) Prion protein expression level alters regional copper, iron and zinc content in the mouse brain. Metallomics 3: 206–214. 21264406 10.1039/c0mt00037j

[18] Bandyopadhyay S, Cahill C, Balleidier A, Huang C, Lahiri DK, et al (2013) Novel 5′ untranslated region directed blockers of iron-regulatory protein-1 dependent amyloid precursor protein translation: implications for down syndrome and Alzheimer's disease. PLoS ONE 8: e65978. 23935819 10.1371/journal.pone.0065978 PMC3729844

[19] Wong BX, Duce JA (2014) The iron regulatory capability of the major protein participants in prevalent neurodegenerative disorders. Front Pharmacol 5: 81. 24795635 10.3389/fphar.2014.00081 PMC4001010

[20] Salazar J, Mena N, Hunot S, Prigent A, Alvarez-Fischer D, et al (2008) Divalent metal transporter 1 (DMT1) contributes to neurodegeneration in animal models of Parkinson's disease. Proc Natl Acad Sci U S A 105: 18578–18583. 19011085 10.1073/pnas.0804373105 PMC2587621

